# Noninferiority of single-incision laparoscopy vs conventional laparoscopy in salpingectomy or salpingotomy for ectopic pregnancy: a meta-analysis

**DOI:** 10.1016/j.xagr.2024.100435

**Published:** 2024-12-15

**Authors:** Greg J. Marchand, Ahmed Massoud, Hollie Ulibarri, Amanda Arroyo, Daniela Gonzalez Herrera, Brooke Hamilton, Kate Ruffley, Mckenna Robinson, Marissa Dominick, Ali Azadi

**Affiliations:** 1 Marchand Institute for Minimally Invasive Surgery, Mesa, AZ (Marchand, Massoud, Ulibarri, Arroyo, Herrera, Hamilton, Ruffley, and Robinson); 2 Faculty of Medicine, Fayoum University, Faiyum, Egypt (Massoud); 3 Midwestern University Chicago College of Osteopathic Medicine, Glendale, AZ (Dominick); 4 College of Medicine, University of Arizona, Phoenix, AZ (Azadi); 5 School of Medicine, Creighton University, Phoenix, AZ (Azadi)

**Keywords:** ectopic pregnancy, laparoscopy, single incision

## Abstract

**OBJECTIVE:**

Ectopic pregnancy is an emergency frequently requiring laparoscopic intervention. This study aimed to determine whether single-incision laparoscopic surgery is a safe and effective treatment method compared with conventional laparoscopic surgery with multiple ports.

**DATA SOURCES:**

This study searched 6 databases from their inception to May 15, 2024, for articles comparing the safety outcomes of single-incision laparoscopic surgery with conventional laparoscopic surgery in managing women with ectopic pregnancy.

**STUDY ELIGIBILITY CRITERIA:**

This study included all studies that evaluated the safety outcomes of single-incision laparoscopic surgery compared with conventional laparoscopic surgery in patients with ectopic pregnancy and included at least 1 of our preselected outcomes. In addition, this study included both randomized controlled trials and observational studies.

**METHODS:**

Review Manager (version 5.4.1) and OpenMetaAnalyst software were used to analyze the extracted data. In addition, this study used odds ratios for dichotomous outcomes, mean difference for continuous outcomes, a fixed effects model for homogeneous outcomes, and a random effects model for heterogeneous outcomes. Furthermore, heterogeneity was evaluated using the *I^2^* and *P* values. After removing duplicates, this study identified 83 studies. Using a 2-step screening process, this study excluded non-English and animal studies and included randomized controlled trials and observational studies that included at least 1 of our preselected outcomes. Ultimately, 12 studies were included in the final synthesis.

**RESULTS:**

Our analysis showed a significant favoring of the single-incision laparoscopic surgery group in the pain visual analog scale score (median difference=−0.57; *P*<.01). However, our study found no statistically significant difference between both procedures in the times of analgesic use (median difference=−0.08; *P*=.19), intraoperative complications (odds ratio=1.17; *P*=.8), postoperative complications (odds ratio=1.02; *P*=.96), conversion to laparotomy (odds ratio=1.40; *P*=.59), bowel injury (odds ratio=1.42; *P*=.8), and postoperative fever (odds ratio=0.52; *P*=.42).

**CONCLUSION:**

The use of single-incision laparoscopic surgery for treating ectopic pregnancy may reduce postoperative pain with similar rates of analgesic use. The incidences of intraoperative and postoperative complications were comparable. Furthermore, the rates of conversion to laparotomy, bowel injury, and postoperative fever were similar between the 2 techniques. Our results seem to show that single-incision laparoscopic surgery is noninferior to conventional laparoscopic surgery for the safe treatment of ectopic pregnancy.

**AJOG Global Reports at a Glance**

**Why was this study conducted?**

Single port laparoscopy is a minimally invasive technique gaining popularity in the treatment of ectopic pregnancy.

Little data exists as to the safety and efficacy of single port laparoscopic techniques versus conventional multiport techniques.

**What are the key findings?**

There seemed to be less postoperative pain in the single port group, and there seemed to be a similar rate of conversion to laparotomy between the two groups.

**What does this study add to what is already known?**

This study adds to the body of evidence that single port laparoscopy is a safe and acceptable treatment for ectopic pregnancy.

## Introduction

Ectopic pregnancy (EP) is a common gynecologic emergency that has increased in frequency over the last decade.^1^ EP occurs when the zygote is implanted outside the normal uterine cavity. The most common site of implantation is the ampullary section of the fallopian tube. However, the remaining parts of the tube, ovaries, cervix, uterine cornu, abdomen, and even previous hysterotomy scars are possible implantation sites.^2^^,^^3^ Patients with EP may be asymptomatic or present with pelvic pain and vaginal bleeding, and the diagnosis may be made with transvaginal ultrasound and/or serum levels of human chorionic gonadotropin (hCG), sometimes performed serially.^4^^,^^5^ Because of the possibility of life-threatening hemorrhage, surgical or medical interruption of the EP at the time of diagnosis is the evidence-based practice.^6^^,^^7^ Medical treatment with intramuscular methotrexate is often contraindicated or refused by patients, and the laparoscopic approach is the preferred surgical management.^8^, ^9^, ^10^ The surgical management may include either salpingotomy or salpingectomy based on several factors, including the extent of tubal damage, the size of the ectopic mass, and the state of the contralateral tube.^8^^,^^10^

Conventional laparoscopic surgery (CLS) has various advantages over traditional laparotomy, including less tissue trauma, less pain, decreased bleeding, shorter postoperative hospitalization, less adhesions, better wound appearance, and quicker return to normal daily activities.^11^^,^^12^ With the recent innovations in minimally invasive procedures in the gynecologic field, single-incision laparoscopic surgery (SILS) was developed to decrease the size and number of ports in the CLS and lessen tissue trauma.^13^^,^^14^ Furthermore, SILS is often associated with better cosmetic outcomes because the multiport trocars are inserted through a single incision in the umbilicus, which, in many cases, creates little or no abdominal scar.^15^ SILS has been found to be a safe and feasible procedure in certain colorectal,^16^ urological,^17^ and gynecologic diseases.^18^^,^^19^ In addition, in studies examining efficacy, SILS showed promising results in the management of EP compared with CLS.^20^ There are limited publications evaluating the safety of SILS vs CLS. This study aimed to investigate the feasibility and safety of SILS compared with CLS in the management of EP.

## Materials and methods

Our study followed the Preferred Reporting Items for Systematic Reviews and Meta-Analyses (PRISMA) statement guidelines.^21^ We conducted a literature search in online databases, including Scopus, Medline, Web of Science, Cochrane Library, PubMed, and ClinicalTrials.gov, using the following search strategy: (“ectopic pregnancy” OR “tubal pregnancy” OR “Tubal Ectopic Pregnancy”) AND (“laparoendoscopic single-site surgery” OR “single port laparoscop*” OR “single-incision laparoscopic surgery” OR “single incision laparoscopic” OR “single site laparoscopy”). We searched from each database's inception to May 15, 2024.

### Studies selection and eligibility criteria

All English studies that evaluated the safety outcomes of SILS compared with CLS in patients with EP were considered for inclusion in our meta-analysis through 2 processes, title and abstract screening and full-text screening. We included studies that investigated the feasibility and safety outcomes of SILS compared with CLS in the management of EP. Our outcomes of interest were pain visual analog scale (VAS) score, times of analgesics use, intraoperative complications, postoperative complications, conversion to laparotomy, bowel injury, and postoperative fever. We included observational studies and randomized controlled studies. We excluded meta-analyses, review articles, single-arm studies, and studies that did not report our selected outcomes.

### Data extraction

For each included study in our meta-analysis, the following demographic and baseline data were collected: sample size, country, age, body mass index, parity, previous abdominal surgery, presence of hemoperitoneum, duration of amenorrhea, gestational age, hCG level, size of the ectopic mass, and results summary. In addition, we extracted data from our selected outcomes, such as pain VAS score, times of analgesics use, intraoperative complications, postoperative complications, conversion to laparotomy, bowel injury, and postoperative fever.

### Quality assessment

We included randomized controlled studies and observational studies in our included articles. We measured the risk of bias in the observational studies using the National Heart, Lung, and Blood Institute (NHLBI) tool.^22^ The randomized controlled studies were assessed using the Cochrane risk of bias tool.^23^

### Statistical methods

We extracted both continuous and dichotomous outcomes. Review Manager (version 5.4.1) and OpenMetaAnalyst software were used to analyze the extracted data. We used an odds ratio (OR) in analyzing the dichotomous outcomes and a mean difference (MD) in analyzing the continuous outcomes. We evaluated the heterogeneity using the *I^2^* and the *P* value. Heterogeneity was identified if *P*<.1 or I^2^>50%. In addition, we conducted a subgroup analysis comparing the prospective and retrospective studies against each other.

## Results

### Summary of the included studies

**Figure 1:**
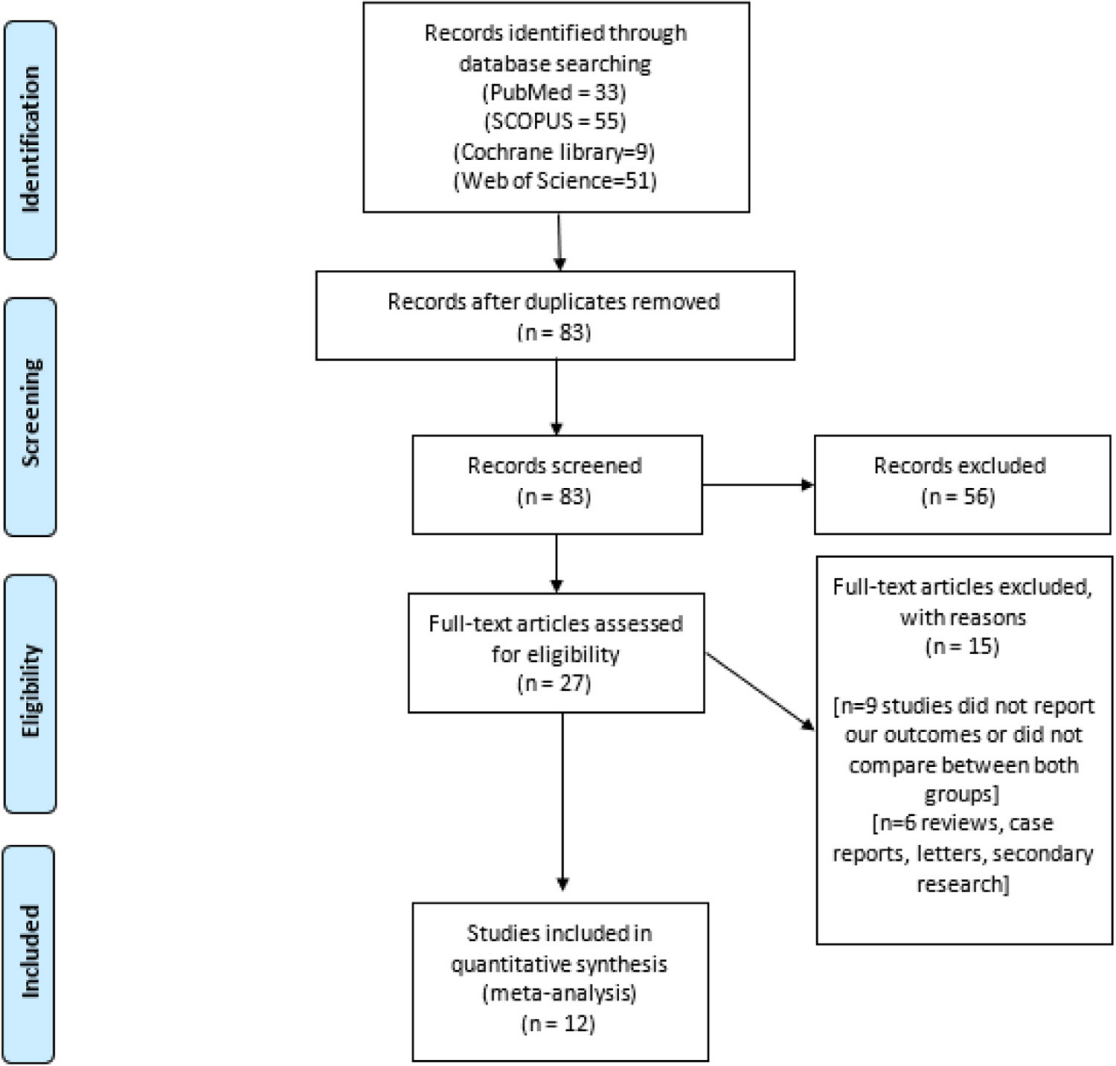
PRISMA diagram flowchart detailing our literature search *PRISMA*, Preferred Reporting Items for Systematic Reviews and Meta-Analyses.

The PRISMA diagram shows our search results in the online databases (Figure 1). The final number of included studies was 12 studies.^13^^,^^20^^,^^24^, ^25^, ^26^, ^27^, ^28^, ^29^, ^30^, ^31^, ^32^, ^33^ A total of 880 women with EP were included. Of these women, 508 underwent CLS, whereas 372 underwent SILS. Table 1, Table 2^13^^,^^20^^,^^24^, ^25^, ^26^, ^27^, ^28^, ^29^, ^30^, ^31^, ^32^, ^33^ present the characteristics of the included studies and patients.

Marchand. Single-incision laparoscopy vs conventional laparoscopy in salpingectomy or salpingotomy for ectopic pregnancy. Am J Obstet Gynecol Glob Rep 2024.

**Table 1:** Characteristics of the included studies and demographic data of the study participants

			Sample size		Age (y)		BMI (kg/m^2^)		Parity	
Study	Summary of results	Country	SILS	CLS	SILS	CLS	SILS	CLS	SILS	CLS
El-Kallaf,^24^ 2018	Total conversion to laparotomy was 3.8% in both groups. Pain scores were significantly lower until 4 h postoperatively in the SILS group than in the CLS group. Single port laparoscopic patients could ambulate within shorter postoperative time, and more patients could ambulate within 3 h postoperative. The mean duration of hospital stay was shorter in the SILS group, with a higher frequency of patients discharged within 24 h postoperatively. Frequency of patients satisfied by wound appearance was higher in the SILS group than in the CLS group.	Kingdom of Saudi Arabia	26	26	24.3±3.6	24.7±3.5	28.6±3.0	28.2±3.5	NR	NR
Karasu and Akselim,^25^ 2019	A total of 53 women, 28 in the CLS group and 25 in the SILS group, participated in the study. There was no difference in demographic characteristics between the 2 groups. There was no difference in terms of variables, including gestational week, beta human chorionic gonadotropin levels, and operation time. No intraoperative complication was observed in either group. The groups exhibited no significant difference regarding additional analgesic requirements or postoperative pain scores. However, pain at the sixth postoperative hour was lower in the SILS group. This effect was not observed at 12 and 24 h.	Turkey	25	28	31.80±5.90	33.40±5.90	26.60±4.10	24.40±3.00	1.75±0.76	2.75±1.98
Kim et al,^27^ 2013	No significance difference was discovered between the groups concerning the adjusted hemoglobin values (*P*=.335). In addition, there was no significant difference in clinical characteristics, intraoperative findings, or operative outcomes.	South Korea	63	71	31.2±5.2	30.4±5.0	21.0±2.1	21.3±2.3	NR	NR
Kim et al,^26^ 2015	There was no statistical difference between the groups in terms of demographic characteristics, operating time, hemoglobin change, return of bowel activity, hospital stay, or complication rate. There was no case of additional trocar use or conversion to laparotomy. Of 5 women with heterotopic pregnancy, 1 underwent SILS, and 3 underwent CLS for tubal pregnancy, which all cases resulted in vaginal delivery without obstetrical complication. However, 1 woman received SILS for cornual pregnancy and had an ongoing pregnancy.	South Korea	26	80	30.70±4.80	30.25±5.16	20.61±1.86	22.50±2.90	0.75±0.75	1.00±0.82
Loh et al,^28^ 2017	Overall, 93 consecutive patients underwent surgery for salpingectomy due to tubal ectopic pregnancy. Of these patients, 33 (group 1) were treated using SILS, and 60 (group 2) were treated using standard CLS. All 33 patients (100%) were treated successfully using SILS, without the need for conversion to CLS or laparotomy. No significant difference was found in mean operative time, length of hospital stay, and patient satisfaction score between the SILS and control groups. No complication was encountered in either group.	Singapore	33	60	30.00±5.95	31.00±5.65	23.80±2.84	25.20±2.08	1.06±1.46	0.77±0.87
Marcelli et al,^20^ 2012	Of note, 97% of patients were treated successfully using SILS. After laparoscopic confirmation of the ectopic pregnancy, salpingectomy was performed with bipolar forceps and scissors. In 1 case, conversion to classic laparoscopy was performed because SILS was not feasible. Compared with the control group, operative time was longer (*P*=.001) but duration of hospitalization was shorter in the SILS group (*P*=.02).	France	37	40	29.3±3.0	28.7±2.8	23.0±4.0	24.0±4.5	1.0±1.1	1.2±1.5
Nasu et al,^29^ 2014	There was no significant difference between the 2 groups regarding the surgical time, blood loss during surgery, or analgesics use after laparoscopic surgery. There was no serious complication and no need for conversion to conventional laparoscopy or laparotomy in both groups.	Japan	6	20	29.3±6.2	31.2±5.4	NR	NR	NR	NR
Seong et al,^30^ 2009	There was no difference between SILS and CLS in terms of mean operative time, mean change from pre- to postoperative hemoglobin level, and mean postoperative hospital day. No complication was encountered in both groups, and there was no need for conversion to conventional laparoscopy in the SILS group.	South Korea	29	30	31.1±5.3	32.6±4.9	NR	NR	NR	NR
Sun et al,^31^ 2018	The characteristics of patients were similar in both groups. There was no statistically significant difference in operative time, estimated blood loss, intraoperative and immediate postoperative complications, and length of hospital stay between both groups. Time to bowel recanalization (*P*<.05) and postoperative visual analog scale for pain scores (*P*<.005) were significantly lower in the SILS group than in the CLS group.	Taiwan	47	65	35.3±5.9	36.9±6.0	NR	NR	NR	NR
Yang et al,^32^ 2016	There was no statistically significant difference in clinical outcomes in either surgical method except for operative time. Operative time of SILS was significantly shorter than CLS for patients with >500 mL of hemoperitoneum.	South Korea	38	45	30.00±2.01	29.14±1.49	22.69±0.87	22.55±0.69	0.62±0.24	0.52±0.20
Yoon et al,^13^ 2011	No significant difference was observed between the 2 groups in terms of mean operative time (*P*=.174), mean difference between pre- and postoperative hemoglobin levels (*P*=.63), or mean postoperative hospital stay (*P*=1.00). No complication was encountered in either group, and there was no conversion to conventional laparoscopy in the SILS group.	South Korea	30	30	30.9±5.4	32.1±5.0	20.6±2.6	20.1±2.2	0.3±0.5	0.2±0.6
Zhang and Zhu,^33^ 2022	The operation time was significantly longer in the SILS group than in the CLS group (*P*<.001). However, blood loss, postoperative exhaustion, pain score, and hospital stay time were significantly lower (*P*<.05 in all cases).	China	12	13	34.5±6.5	36.7±7.2	22.6±3.6	21.7±4.1	NR	NR

Data are presented as mean±standard deviation.

*BMI*, body mass index; *CLS*, conventional laparoscopic surgery; *NR*, not reported; *SILS*, single-incision laparoscopic surgery.

**Table 2:** Baseline characteristics of the included participants

Study	Previous abdominal surgery		Presence of hemoperitoneum		Duration of amenorrhea (d)		Gestational age (wk)		hCG level (mIU/mL)		Size of ectopic mass (cm)	
	SILS	CLS	SILS	CLS	SILS	CLS	SILS	CLS	SILS	CLS	SILS	CLS
El-Kallaf,^24^ 2018	NR	NR	12 (46.2)	15 (57.7)	NR	NR	7.1±1.1	6.8±1.0	NR	NR	NR	NR
Karasu and Akselim,^25^ 2019	7 (28.0)	5 (17.8)	25 (100.0)	28 (100.0)	NR	NR	8.5±1.5	7.0±1.0	5685.6±5929.6	4894.9±3074.6	4.7±2.3	4.6±1.4
Kim et al,^27^ 2013	8 (12.7)	11 (15.5)	NR	NR	53.3±11.5	50.4±10.3	NR	NR	NR	NR	4.0±0.9	3.8±0.8
Kim et al,^26^ 2015	NR	NR	18 (69.2)	53 (74.6)	40.25±10.34	52.75±15.90	NR	NR	NR	NR	NR	NR
Loh et al,^28^ 2017	11 (33.3)	21 (35.0)	NR	NR	NR	NR	NR	NR	9657.30±11734.00	11,053.10±22,350.00	3.48±1.53	3.86±1.16
Marcelli et al,^20^ 2012	NR	NR	28 (75.7)	30 (75.0)	NR	NR	7.4±3.0	7.3±3.0	3500.0±1100.0	3650.0±1200.0	NR	NR
Nasu et al,^29^ 2014	NR	NR	2 (33.0)	1 (5.0)	NR	NR	6.3±1.2	7.2±1.3	NR	NR	NR	NR
Seong et al,^30^ 2009	NR	NR	NR	NR	NR	NR	NR	NR	NR	NR	NR	NR
Sun et al,^31^ 2018	NR	NR	NR	NR	NR	NR	NR	NR	NR	NR	NR	NR
Yang et al,^32^ 2016	6 (15.0)	7 (14.5)	38 (100.0)	45 (100.0)	NR	NR	7.00±0.46	7.75±0.68	NR	NR	NR	NR
Yoon et al,^13^ 2011	3 (10.0)	7 (23.3)	9 (30.0)	11 (36.7)	52.0±14.0	48.4±9.9	NR	NR	5442.0±7802.0	6921.0±10,366.0	3.4±1.6	3.2±1.3
Zhang and Zhu,^33^ 2022	NR	NR	NR	NR	NR	NR	NR	NR	NR	NR	NR	NR

Data are presented as number (percentage) or mean±standard deviation.

*CLS*, conventional laparoscopic surgery; *hCG*, human chorionic gonadotropin; *NR*, not reported; *SILS*, single-incision laparoscopic surgery.

### Results of risk of bias assessment

The observational studies were assessed using the NHLBI tool,^22^ yielding an overall score of 9.0 of 14.0 in cohort studies and 10.1 of 12.0 in the case-control studies. Complete details can be found in Supplementary Tables S1 and S2. Concerning the randomized controlled studies,^24^^,^^33^ the overall risk of bias assessment according to the Cochrane tool^23^ was a moderate risk of bias, as demonstrated in Supplementary Table S3.

## Analysis of outcomes

### Visual analog scale pain score on postoperative day 1

**Figure 2:**
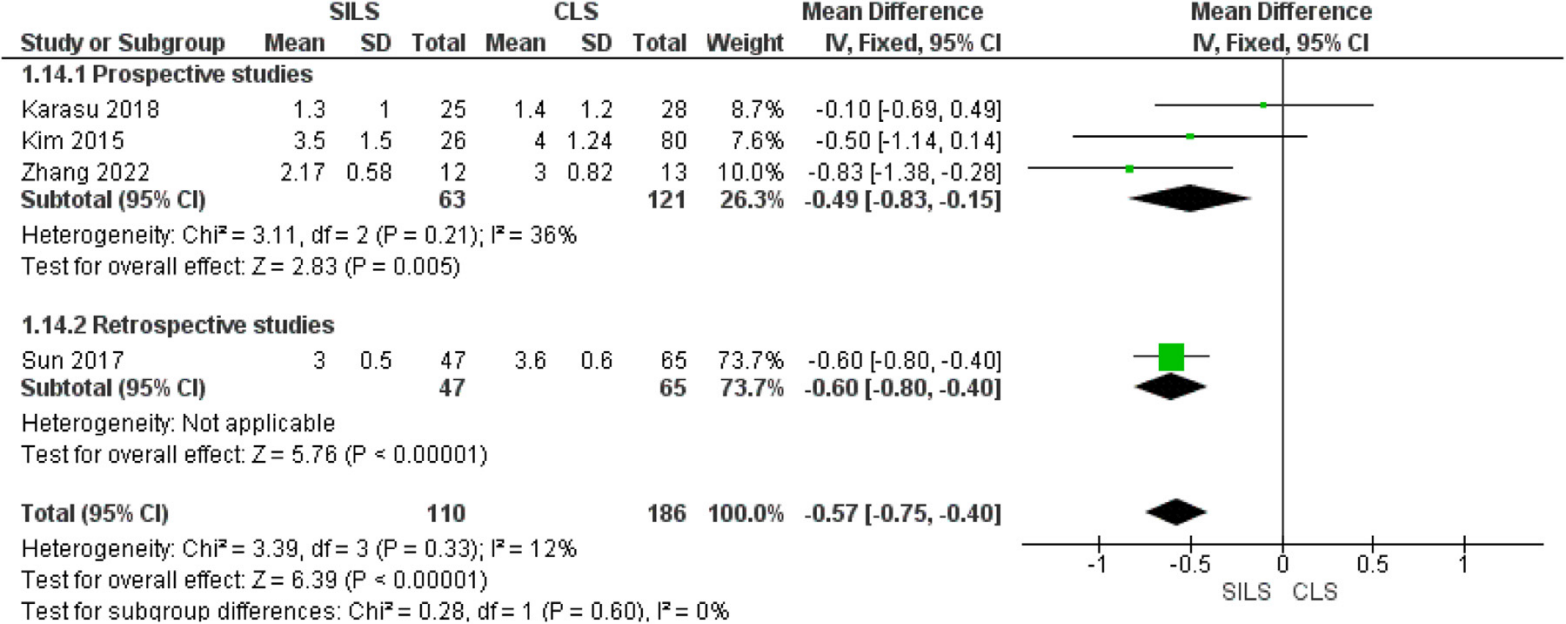
Meta-analysis of the VAS pain score on postoperative day 1 *CI*, confidence interval; *CLS*, conventional laparoscopic surgery; *SD*, standard deviation; *SILS*, single-incision laparoscopic surgery; *VAS*, visual analog scale.

Of note, 4 studies reported the VAS pain score on postoperative day 1.^25^^,^^26^^,^^31^^,^^33^ The subgroup analysis for prospective studies showed a considerably lower pain VAS score in patients who underwent SILS by MD= −0.49 [−0.83, −0.15], (P = 0.005). In addition, Sun et al^33^ in the retrospective subgroup favored the SILS group (*P*<.001). Our combined analysis showed a significantly decreased VAS score in patients who underwent SILS compared with those who underwent CLS (MD=−0.57; 95% CI, −0.75 to −0.40; *P*<.01). We observed homogeneity in this outcome (*P*=.33; *I²*=12%) (Figure 2).

Marchand. Single-incision laparoscopy vs conventional laparoscopy in salpingectomy or salpingotomy for ectopic pregnancy. Am J Obstet Gynecol Glob Rep 2024.

### Number of times intravenous analgesia was required postoperatively

**Figure 3:**
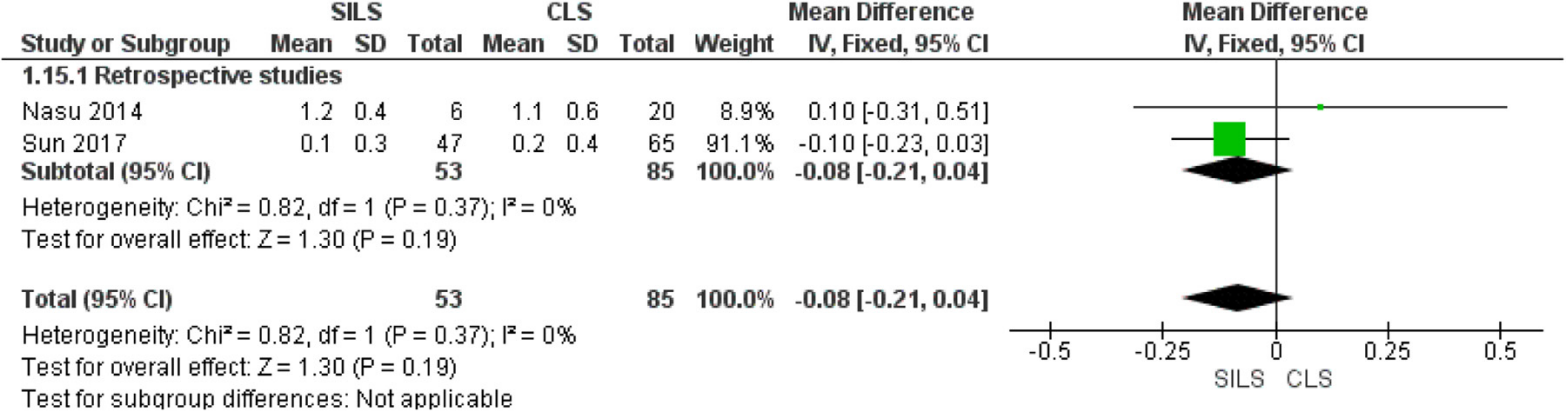
Meta-analysis of the number of times intravenous analgesia was required postoperatively *CI*, confidence interval; *CLS*, conventional laparoscopic surgery; *SD*, standard deviation; *SILS*, single-incision laparoscopic surgery.

Of note, 2 retrospective studies reported this outcome.^29^^,^^31^ We found no significant variation between both procedures (MD=−0.08; 95% CI, −0.21 to 0.04; *P*=.19). We observed homogeneity in this outcome (*P*=.37; *I²*=0%) (Figure 3).

Marchand. Single-incision laparoscopy vs conventional laparoscopy in salpingectomy or salpingotomy for ectopic pregnancy. Am J Obstet Gynecol Glob Rep 2024.

### Any intraoperative complication

**Figure 4:**
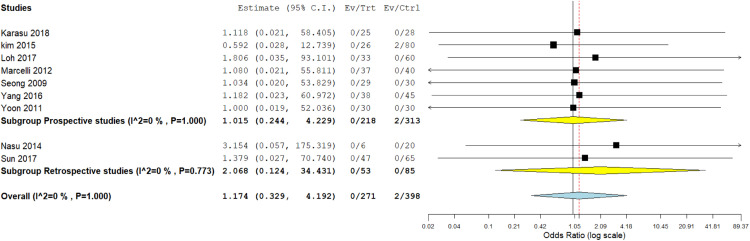
Meta-analysis of the occurrence of any intraoperative complication *CI*, confidence interval.

Most studies reported intraoperative complications.^13^^,^^20^^,^^25^^,^^26^^,^^28^, ^29^, ^30^, ^31^, ^32^ The prospective subgroup analysis showed no significant difference between both techniques (OR, 1.015; 95% CI, 0.244–4.229; *P*=.984). Similarly, the retrospective subgroup analysis showed a similar incidence of intraoperative complications (OR, 2.068; 95% CI, 0.124–34.431; *P*=.613). Pooled analysis showed similar amounts of intraoperative complications in both procedures (OR, 1.174; 95% CI, 0.329–4.192; *P*=.805). The pooled data were homogeneous (*P*=1.00; *I²*=0%) (Figure 4).

Marchand. Single-incision laparoscopy vs conventional laparoscopy in salpingectomy or salpingotomy for ectopic pregnancy. Am J Obstet Gynecol Glob Rep 2024.

### Any postoperative complication

**Figure 5:**
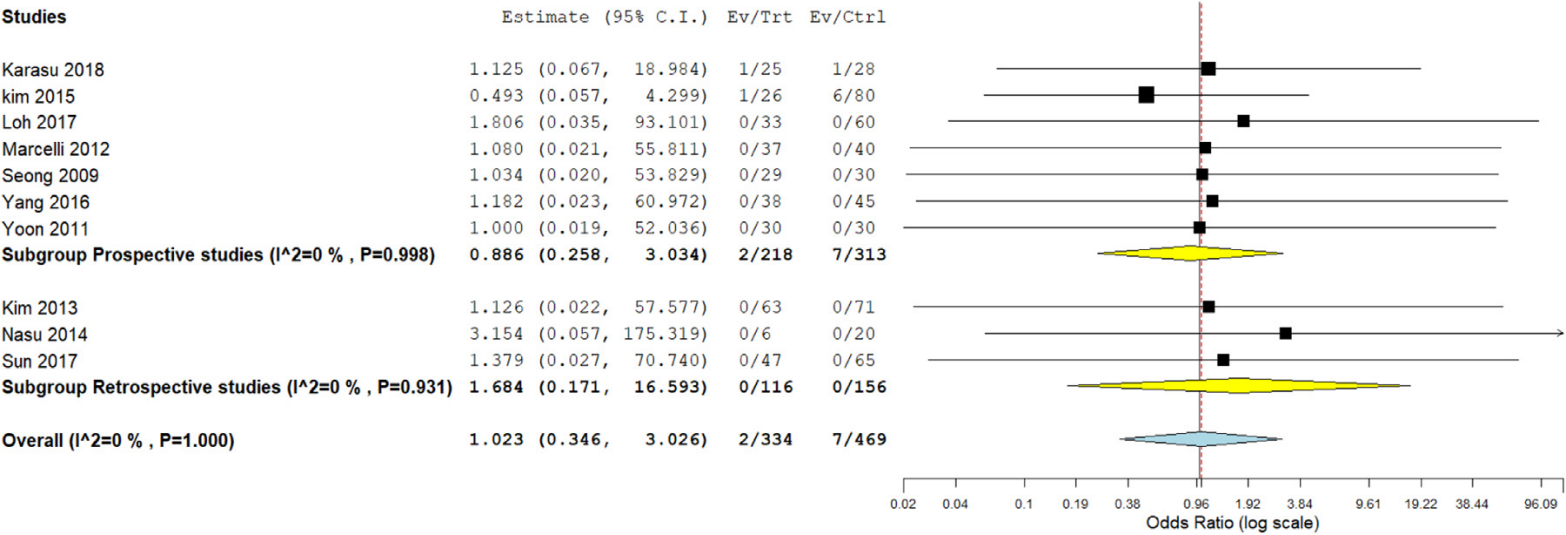
Meta-analysis of the occurrence of any postoperative complication *CI*, confidence interval.

Of note, 10 studies^13^^,^^20^^,^^25^, ^26^, ^27^, ^28^, ^29^, ^30^, ^31^, ^32^ reported the incidence of postoperative complications in patients in both cohorts. Concerning the prospective studies subgroup, both procedures were associated with similar postoperative complications (OR, 0.886; 95% CI, 0.258–3.034; *P*=.847). Regarding the retrospective subgroup analysis, we also observed similar postoperative complications among patients in both groups (OR, 1.684; 95% CI, 0.171–16.593; *P*=.655). In addition, pooled analysis showed no considerable variation between the 2 procedures (OR, 1.023; 95% CI, 0.346–3.026; *P*=.967). The data were homogeneous (*P*=1.00; *I²*=0%) (Figure 5).

Marchand. Single-incision laparoscopy vs conventional laparoscopy in salpingectomy or salpingotomy for ectopic pregnancy. Am J Obstet Gynecol Glob Rep 2024.

### Conversion to laparotomy

**Figure 6:**
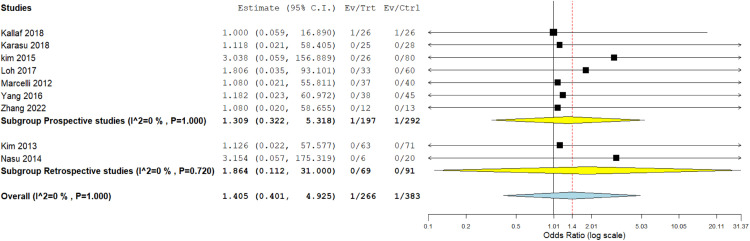
Meta-analysis of the rate of conversion to laparotomy *CI*, confidence interval.

The incidence of conversion to laparotomy was measured in 9 studies.^20^^,^^24^, ^25^, ^26^, ^27^, ^28^, ^29^^,^^32^^,^^33^ Regarding the prospective subgroup, we analyzed data from 7 studies, which showed no difference between both techniques (OR, 1.309; 95% CI, 0.322–5.318; *P*=.706). Concerning the retrospective studies, we also found a similar incidence of conversion to laparotomy in both procedures (OR, 1.864; 95% CI, 0.112–31.000; *P*=0.664). The overall OR of both subgroups showed a comparable incidence of conversion to laparotomy (OR, 1.405; 95% CI, 0.401–4.925; *P*=.596). We found homogeneity among data in this outcome (*P*=1.00; *I²*=0%) (Figure 6).

Marchand. Single-incision laparoscopy vs conventional laparoscopy in salpingectomy or salpingotomy for ectopic pregnancy. Am J Obstet Gynecol Glob Rep 2024.

### Bowel injury

**Figure 7:**
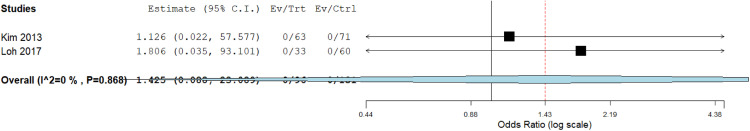
Meta-analysis of the incidence of bowel injury *CI*, confidence interval; *CLS*, conventional laparoscopic surgery; *SILS*, single-incision laparoscopic surgery.

Of note, 2 studies^27^^,^^28^ evaluated the risk of bowel injury among patients in both cohorts. Our analysis demonstrated a similar incidence of bowel injury in both procedures (OR, 1.425; 95% CI, 0.088–23.890; *P*=.803). We observed no heterogeneity among data (*P*=.86; *I²*=0%) (Figure 7).

Marchand. Single-incision laparoscopy vs conventional laparoscopy in salpingectomy or salpingotomy for ectopic pregnancy. Am J Obstet Gynecol Glob Rep 2024.

### Postoperative fever

**Figure 8:**
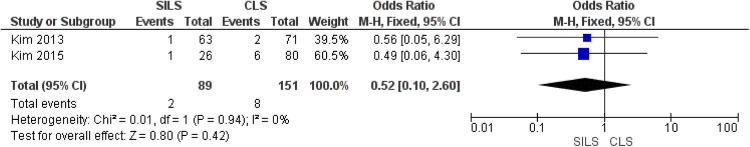
Meta-analysis of the incidence of postoperative fever *CI*, confidence interval; *CLS*, conventional laparoscopic surgery; *SILS*, single-incision laparoscopic surgery.

The incidence of postoperative fever was comparable in both procedures with a combined OR of 0.52 (95% CI, 0.10–2.60; *P*=.42). Data were homogeneous (*P*=.94; *I²*=0%) (Figure 8).

Marchand. Single-incision laparoscopy vs conventional laparoscopy in salpingectomy or salpingotomy for ectopic pregnancy. Am J Obstet Gynecol Glob Rep 2024.

## Discussion

The objective of this study was to estimate and compare the safety results of SILS with CLS in EP treatment. The conclusions derived from the results offer useful knowledge regarding the advantages and disadvantages that may be inherent in the 2 techniques of surgery. Among all assessed parameters, the most notable result was the decreased level of postoperative pain in patients who underwent SILS. This implies that a single incision may cause less discomfort after the surgery compared to the multiple incisions in CLS, which may explain the aspects of improved patient satisfaction and quicker recovery.^34^

Despite the reduced pain scores observed in the SILS group, the administration of postoperative analgesics did not vary between the groups. This means that although the patients complained of less pain during SILS, the clinical approach to addressing pain may be similar between the 2 techniques.^35^ In addition, this study did not reveal any increase in intraoperative and postoperative complications in cases of SILS compared with those of CLS. This shows that SILS is less invasive regarding incisions but may not worsen patient safety or increase the probability of complications during or after the operation.

In addition, the percentage of conversions to laparotomy because of complications or defective visualization was similar in the 2 groups, which presents SILS as an effective option for CLS. Moreover, the incidences of bowel injury and postoperative fever showed no significant difference between patients in both groups.

Although our analysis contained more than double the amount of studies, our findings were largely in line with the most recent meta-analysis on this topic, Gasparri et al,^36^ published in 2018. This study reported no difference between conventional laparoscopy and single-site surgery concerning the length of operative time, hospitalization, change in hemoglobin levels, transfusion rate, and complications. This study did not include pain scores as an outcome but included only 5 studies comparing single and multiport treatments.

Regarding the importance of pain scores, the effect of SILS on postoperative pain varies in the literature. Of note, 1 systematic review, Murji et al,^37^ observed VAS scores 24 hours postoperatively and beyond and found no statistically significant difference in pain between the SILS and CLS for adnexal masses. However, in many of our included studies, such as the study of Kim et al,^26^ VAS pain scores were significantly lower on the first postoperative day in the SILS group. This raises the possibility that assessing postoperative pain from the perspective of the patients may introduce some bias, as there is no definitive way to quantify pain felt. Of note, 1 study that attempted to solve this issue was Karasu and Akselim.^25^ Their method was to use VAS pain scales in combination with the assessment of the number of times analgesics were needed. Overall, in our study, we noted that the patients in the SILS group generally reported less pain the day after the operation. In addition, there was no statistically meaningful variation between the 2 procedures in the actual requirement for more analgesia.

One of our included RCTs, Zhang and Zhu,^33^ compared SILS and CLS in patients with EP. The findings suggested that SILS provided superior intraoperative and postoperative conditions, reduced postoperative pain, and faster recovery times. This is in agreement with the other included randomized controlled trial (RCT), El-Kallaf,^24^ which also showed faster ambulation, hospital discharge, and higher satisfaction in the single port group. However, this 2018 study had an unusually high conversion to laparotomy rate of 3.8% across both groups.^24^

Most studies on this topic have focused on the use of SILS in hemodynamically stable women with tubal pregnancies,^13^^,^^35^^,^^38^ which limits their clinical applicability given that hemodynamic instability and hemoperitoneum are common and the type of EP is often undiagnosed before surgery. Kim et al^26^ addressed this limitation by including all patients scheduled for surgery for EP, regardless of their type of EP, hemodynamic status, or history of abdominal surgery. Their findings revealed no considerable variation in operative results or complication rates between the SILS and CLS groups. Thus, they concluded that SILS is a feasible and safe option not only for hemodynamically stable women with tubal pregnancies but also for those who are hemodynamically unstable or have other types of EPs. These findings were seemingly confirmed by Karasu and Akselim^25^ in 2019, where they compared the 2 procedures in the presence of severe hemoperitoneum and did not find any intraoperative complications, despite a total of 53 procedures between the 2 groups. However, Karasu and Akselim^25^ noted that postoperative pain scores were notably lower in the SILS group when measured 12 hours postoperatively.

### Limitation

The main limitation of our study was the sample size, which was relatively small. Having only 2 RCTs on this topic forced us to include other study designs, which lowered the strength of the findings. In addition, secondary to sample size, we were unable to subgroup patients by salpingectomy and salpingotomy or by study design. Finally, we did not exclude any studies based on surgeon skill level or experience, as this information was not given in most included studies. It is possible that there were significant differences in the skill or experience levels of the surgeons in the included studies, which skewed our results.

## Conclusion

SILS seems to reduce postoperative pain compared with CLS in the treatment of EP. Despite this, the use of analgesics was similar between the 2 groups. Intraoperative and postoperative complications were comparable between SILS and CLS, thereby affirming that SILS does not seem to increase the risk of complications. In addition, the rates of conversion to laparotomy, bowel injury, and postoperative fever were similar between the 2 techniques. These results seem to show that SILS is noninferior to CLS for the safe treatment of EP.

## CRediT authorship contribution statement

**Greg J. Marchand:** Writing – original draft, Supervision, Project administration, Methodology, Investigation, Data curation, Conceptualization. **Ahmed Massoud:** Writing – review & editing, Formal analysis, Data curation. **Hollie Ulibarri:** Formal analysis, Data curation. **Amanda Arroyo:** Supervision, Data curation. **Daniela Gonzalez Herrera:** Data curation. **Brooke Hamilton:** Validation, Data curation. **Kate Ruffley:** Data curation. **Mckenna Robinson:** Data curation. **Marissa Dominick:** Visualization, Validation, Investigation, Formal analysis. **Ali Azadi:** Writing – review & editing, Supervision.

## Appendix Supplementary materials

Image, application 1

## Footnotes

Supplementary material associated with this article can be found in the online version at doi:10.1016/j.xagr.2024.100435.
