# Supplementary material for: Noninferiority of single-incision laparoscopy vs conventional laparoscopy in salpingectomy or salpingotomy for ectopic pregnancy: a meta-analysis
Source: AJOG Glob Rep. 2024 Dec 15;5(1):100435. doi: 10.1016/j.xagr.2024.100435 (PMC11773238; doi:10.1016/j.xagr.2024.100435)
Supplement: Supplementary file 1 [file mmc1.docx]

**Supplementary Table 1:** Quality assessment of the included cohort observational studies.

| Study name | Seong 2009 | Sun 2017 |
| --- | --- | --- |
| 1.Was the research question or objective in this paper clearly stated? | Yes | Yes |
| 2.Was the study population clearly specified and defined? | Yes | Yes |
| 3.Was the participation rate of eligible persons at least 50%? | Yes | Yes |
| 4.Were all the subjects selected or recruited from the same or similar populations (including the same time period)? Were inclusion and exclusion criteria for being in the study prespecified and applied uniformly to all participants? | NA | Yes |
| 5. Was a sample size justification, power description, or variance and effect estimates | No | Yes |
| 6.For the analyses in this paper, were the exposure (s) of interest measured prior to the outcome(s) being measured? | Yes | Yes |
| 7. Was the timeframe sufficient so that one could reasonably expect to see an association between exposure and outcome if it existed? | Yes | Yes |
| 8. For exposures that can vary in amount or level, did the study examine different levels of the exposure as related to the outcome (e.g., categories of exposure, or exposure measured as continuous variable)? | No | No |
| 9. Were the exposure measures (independent variables) clearly defined, valid, reliable, and implemented consistently across all study participants? | No | Yes |
| 10. Was the exposure(s) assessed more than once over time? | NA | NA |
| 11. Were the outcome measures (dependent variables) clearly defined, valid, reliable, and implemented consistently across all study participants? | Yes | Yes |
| 12. Were the outcome assessors blinded to the exposure status of participants? | No | No |
| 13. Was loss to follow-up after baseline 20% or less? | * | Yes |
| 14. Were key potential confounding variables measured and adjusted statistically for their impact on the relationship between exposure(s) and outcome(s)? | Yes | Yes |
| Total score (out of 14) | 7 | 11 |

Key: 1 = Yes, 0 = No, * = Not reported, N/A = Not applicable.

**Supplementary Table 2:** Quality assessment of the included case control observational studies.

|  | Karasu 2018 | Kim 2013 | Kim 2015 | Loh 2017 | Marcelli 2012 | Nasu 2014 | Yang 2016 | Yoon 2011 |
| --- | --- | --- | --- | --- | --- | --- | --- | --- |
| 1. Was the research question or objective in this paper clearly stated and appropriate? | 1 | 1 | 1 | 1 | 1 | 1 | 1 | 1 |
| 2.Was the study population clearly specified and defined? | 1 | 1 | 1 | 1 | 1 | 1 | 1 | 1 |
| 3. Did the authors include a sample size justification? | 1 | 1 | 1 | 0 | 1 | 1 | 1 | 1 |
| 4. Were controls selected or recruited from the same or similar population that gave rise to the cases (including the same timeframe)? | 1 | 1 | 1 | * | * | 1 | 0 | 1 |
| 5. Were the definitions, inclusion and exclusion criteria, algorithms or processes used to identify or select cases and controls valid, reliable, and implemented consistently across all study participants? | 1 | 0 | 0 | 0 | 0 | 0 | 1 | 1 |
| 6. Were the cases clearly defined and differentiated from controls? | 1 | 1 | 1 | 1 | 1 | 1 | 1 | 1 |
| 7. If less than 100 percent of eligible cases and/or controls were selected for the study, were the cases and/or controls randomly selected from those eligible? | 1 | 1 | 1 | 1 | 1 | 1 | 1 | 1 |
| 8. Was there use of concurrent controls? | 1 | 1 | 1 | 1 | 1 | 1 | 1 | 1 |
| 9. Were the investigators able to confirm that the exposure/risk occurred prior to the development of the condition or event that defined a participant as a case? | 1 | 1 | 1 | 1 | 1 | 1 | 1 | 1 |
| 10. Were the measures of exposure/risk clearly defined, valid, reliable, and implemented consistently (including the same time period) across all study participants? | 0 | 0 | 1 | 1 | 1 | 0 | 0 | 0 |
| 11. Were the assessors of exposure/risk blinded to the case or control status of participants? | 1 | 1 | 1 | 1 | 1 | 1 | 1 | 1 |
| 12.  Were key potential confounding variables measured and adjusted statistically in the analyses? If matching was used, did the investigators account for matching during study analysis? | 1 | 1 | 0 | 1 | 1 | 1 | 0 | 1 |
| Total score (out of 12) | 11/12 | 11/12 | 10/12 | 9/12 | 10/12 | 10/12 | 9/12 | 11/12 |

Key: 1 = Yes, 0 = No, * = Not reported, N/A = Not applicable.

**Supplementary Table 3:** Quality assessment of the included randomized controlled studies.

| Study | Randomization | Allocation concealment | Blinding of participants and personnel | Blinding of outcome assessment | Attrition bias | Selective reporting |
| --- | --- | --- | --- | --- | --- | --- |
| El-kallaf 2018 | low | low | high | high | low | low |
| Zhang 2022 | low | High | High | Unclear | Low | low |
